# Supplementary material for: Synthesis, Stability, and Antidiabetic Activity Evaluation of (−)-Epigallocatechin Gallate (EGCG) Palmitate Derived from Natural Tea Polyphenols
Source: Molecules. 2021 Jan 13;26(2):393. doi: 10.3390/molecules26020393 (PMC7828495; doi:10.3390/molecules26020393)
Supplement: Supplementary file 1 [file molecules-26-00393-s001.pdf]

Supporting Information

Synthesis, stability and antidiabetic activity evaluation of EGCG palmitate derived from natural tea polyphenols

Bingbing Liu[1,2], Zhengzhong Kang[1], Weidong Yan[1*]

1. *Department of Chemistry, Zhejiang University, Hangzhou, 310027, China*
2. *State Key Laboratory of Environmental and Biological Analysis, Hong Kong Baptist University, Kowloon Tong, Kowloon, Hong Kong SAR, China*

∗Corresponding author

yanweidong@zju.edu.cn (Weidong Yan). Tel.: +86 571 8795 1430. Fax: +86 571 87951895.

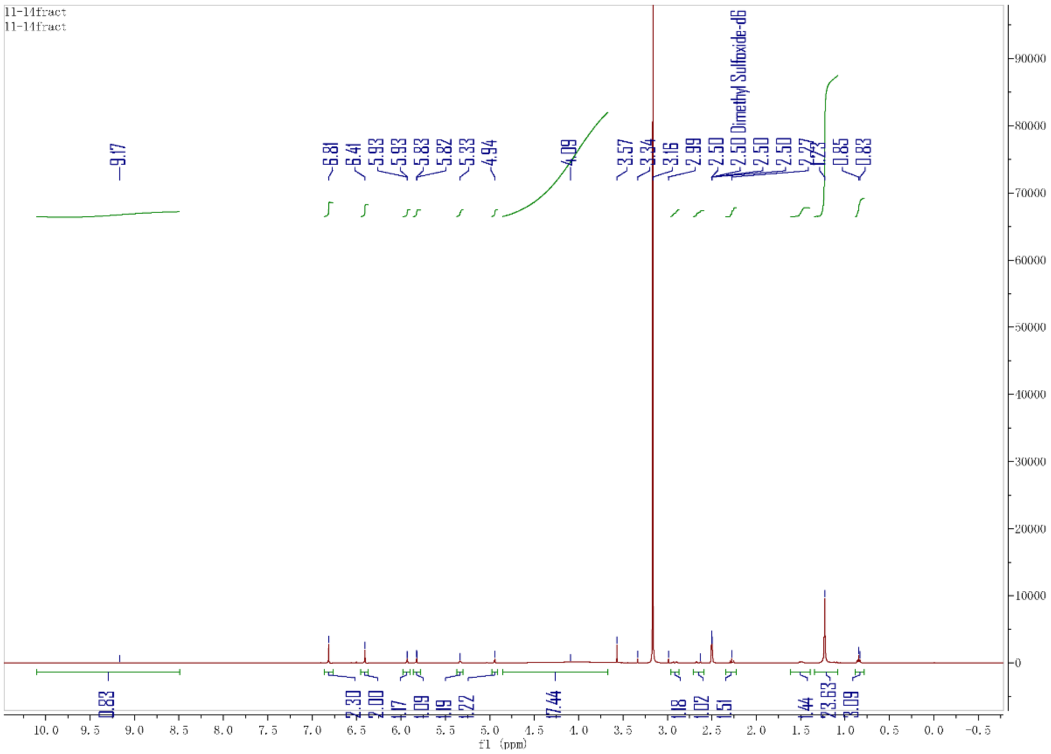

Figure S1. ^{1}H NMR spectrum of PEGCG.

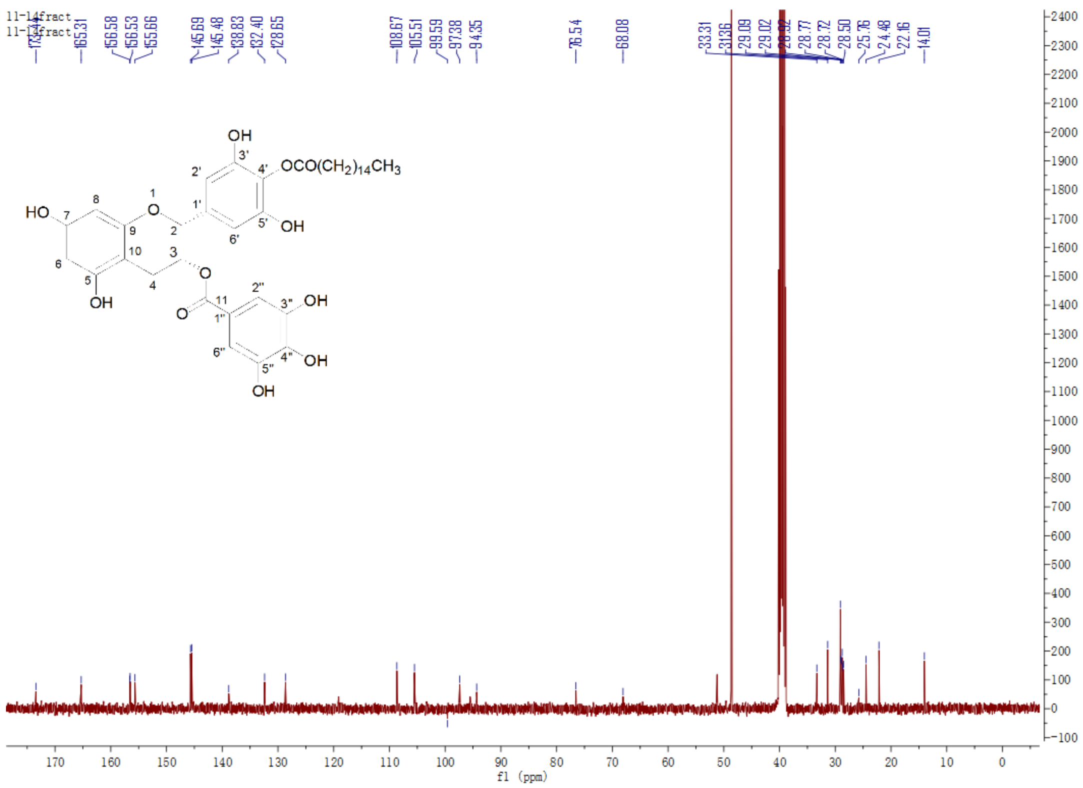

Figure S2. ^{13}C NMR spectrum of PEGCG.

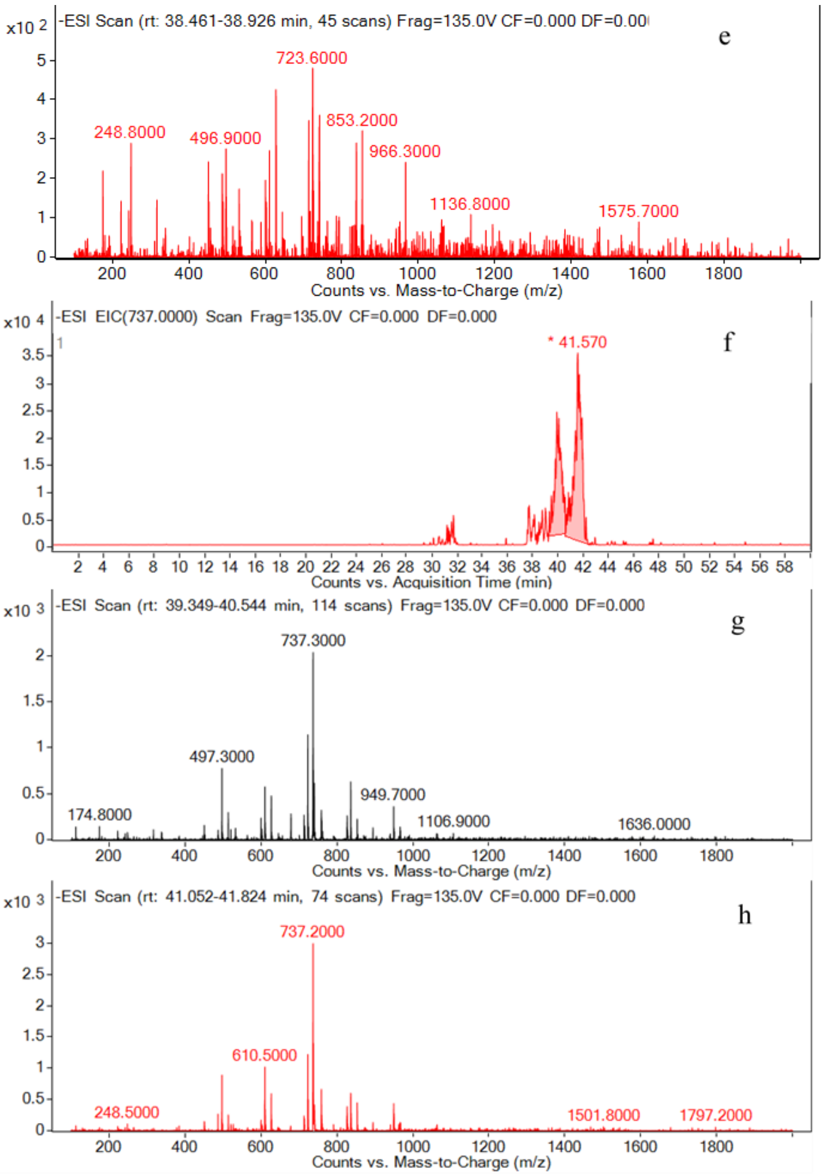

Figure S3. HPLC-MS analysis of byproducts under the optimum condition, (e) mass spectra of peak 4; (f) selective ion chromatogram of m/z 737; (g) mass spectra of peak 5; (h) mass spectra of peak 6.

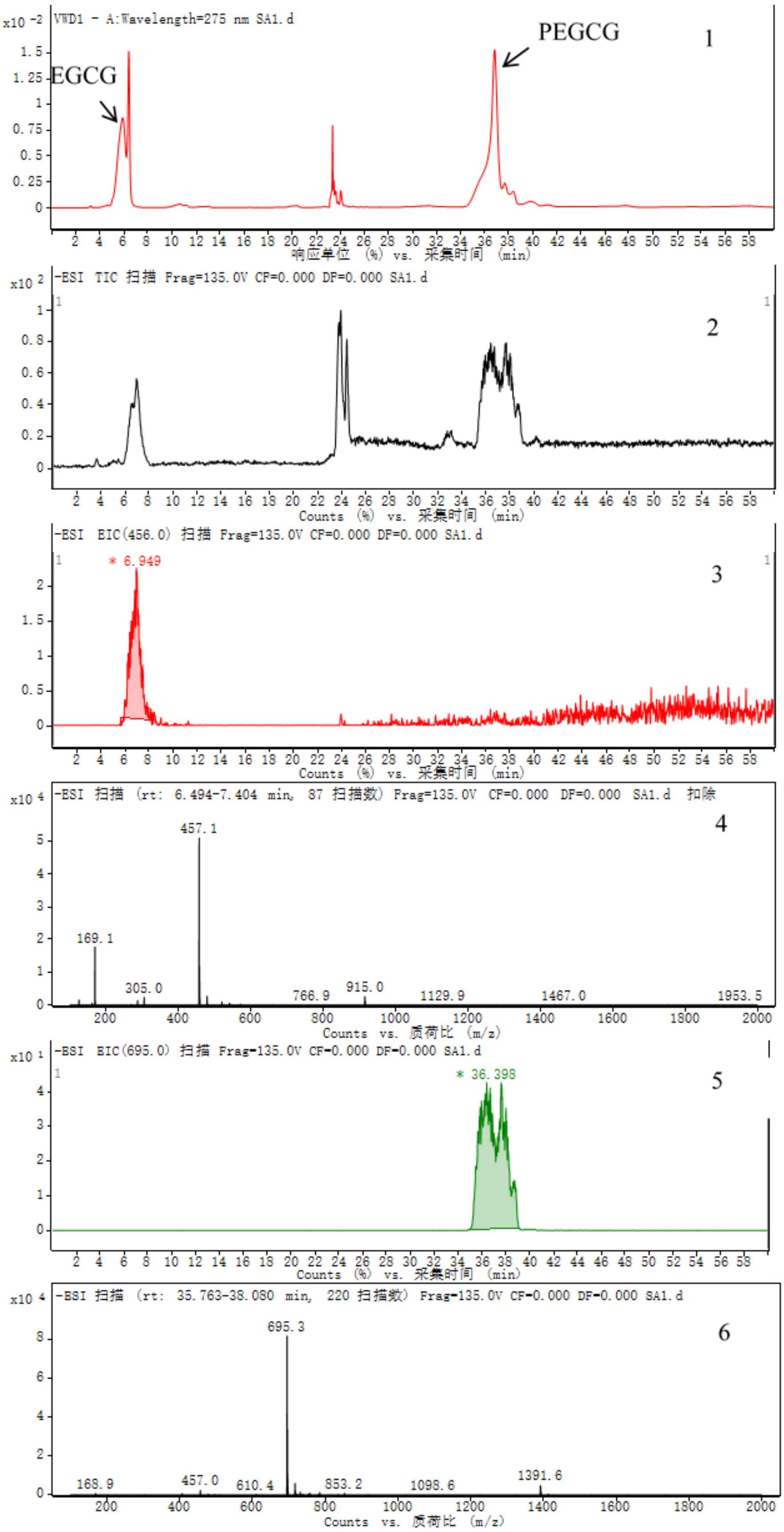

VWD1 - A:Wavelength=275 nm SA1.d
EGCG
PEGCG
1
响应单位 (%) vs. 采集时间 (min)
-ESI TIC 扫描 Frag=135.0V CF=0.000 DF=0.000 SA1.d
2
Counts (%) vs. 采集时间 (min)
-ESI EIC(456.0) 扫描 Frag=135.0V CF=0.000 DF=0.000 SA1.d
* 6.949
3
Counts (%) vs. 采集时间 (min)
-ESI 扫描 (rt: 6.494-7.404 min, 87 扫描数) Frag=135.0V CF=0.000 DF=0.000 SA1.d 扣除
4
457.1
169.1
305.0
766.9
915.0
1129.9
1467.0
1953.5
Counts vs. 质荷比 (m/z)
-ESI EIC(695.0) 扫描 Frag=135.0V CF=0.000 DF=0.000 SA1.d
* 36.398
5
Counts (%) vs. 采集时间 (min)
-ESI 扫描 (rt: 35.763-38.080 min, 220 扫描数) Frag=135.0V CF=0.000 DF=0.000 SA1.d
6
695.3
168.9
457.0
610.4
853.2
1098.6
1391.6
Counts vs. 质荷比 (m/z)

Figure S4. HPLC-MS analysis of product under the condition of 1 molar ratio of palmitoyl chloride, (1) chromatogram of the product; (2) total ion chromatogram; (3) selective ion chromatogram of EGCG (m/z 457); (4) mass spectra of EGCG (Mw 458.4); (5) selective ion chromatogram of PEGCG (m/z 695); (6) mass spectra of PEGCG (Mw 696.8).

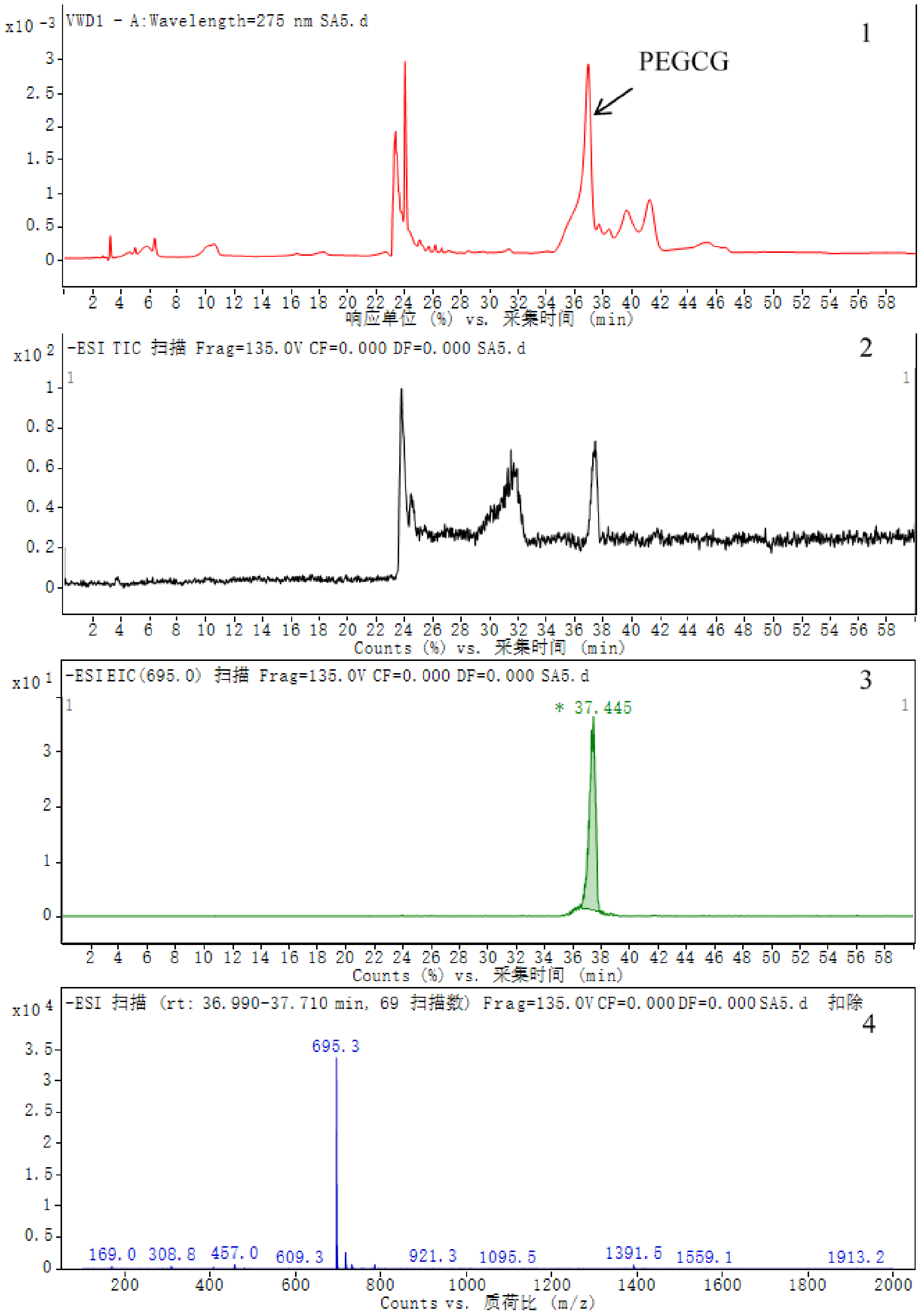

Figure S5. HPLC-MS analysis of product under the condition of 3 molar ratio of palmitoyl chloride, (1) chromatogram of the product; (2) total ion chromatogram; (3) selective ion chromatogram of PEGCG (m/z 695); (4) mass spectra of PEGCG (Mw 696.8).

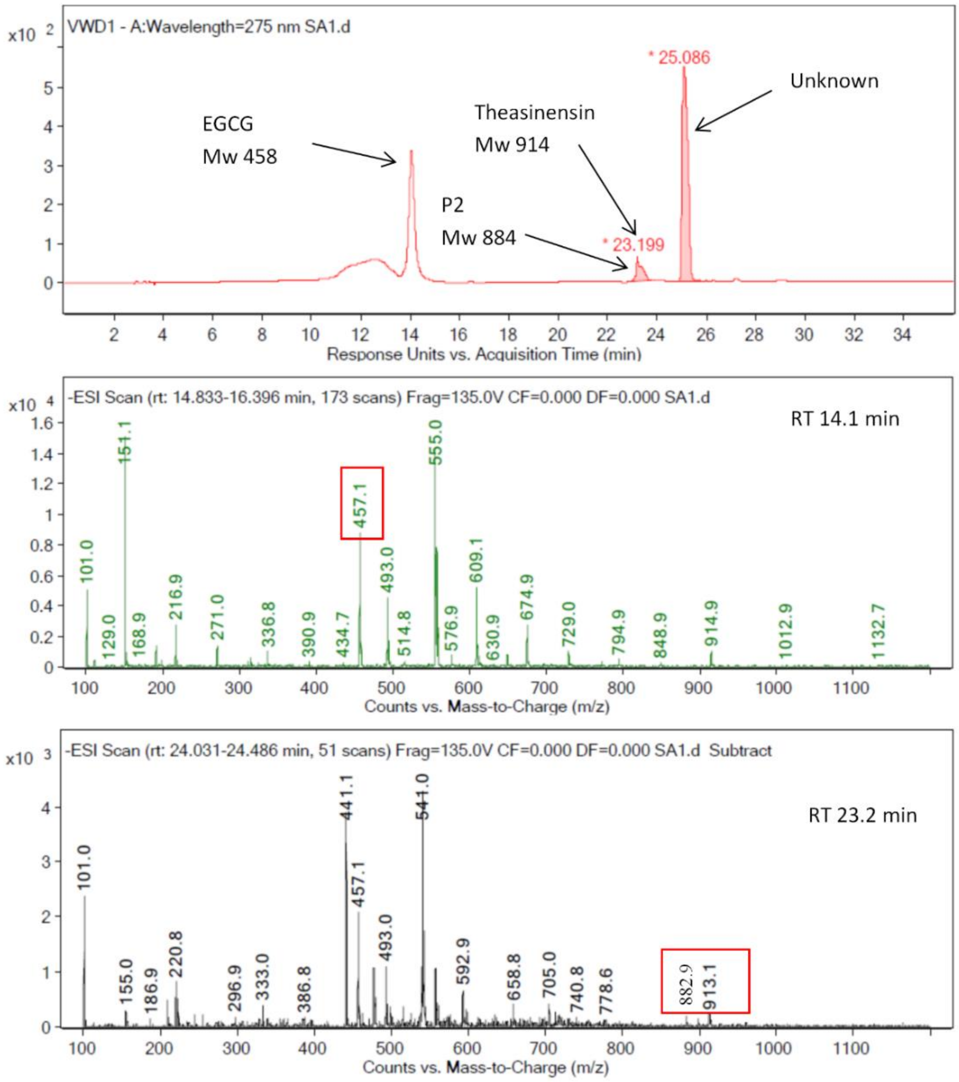

Figure S6. Identification of the oxidation product of EGCG under alkaline condition by HPLC-MS.

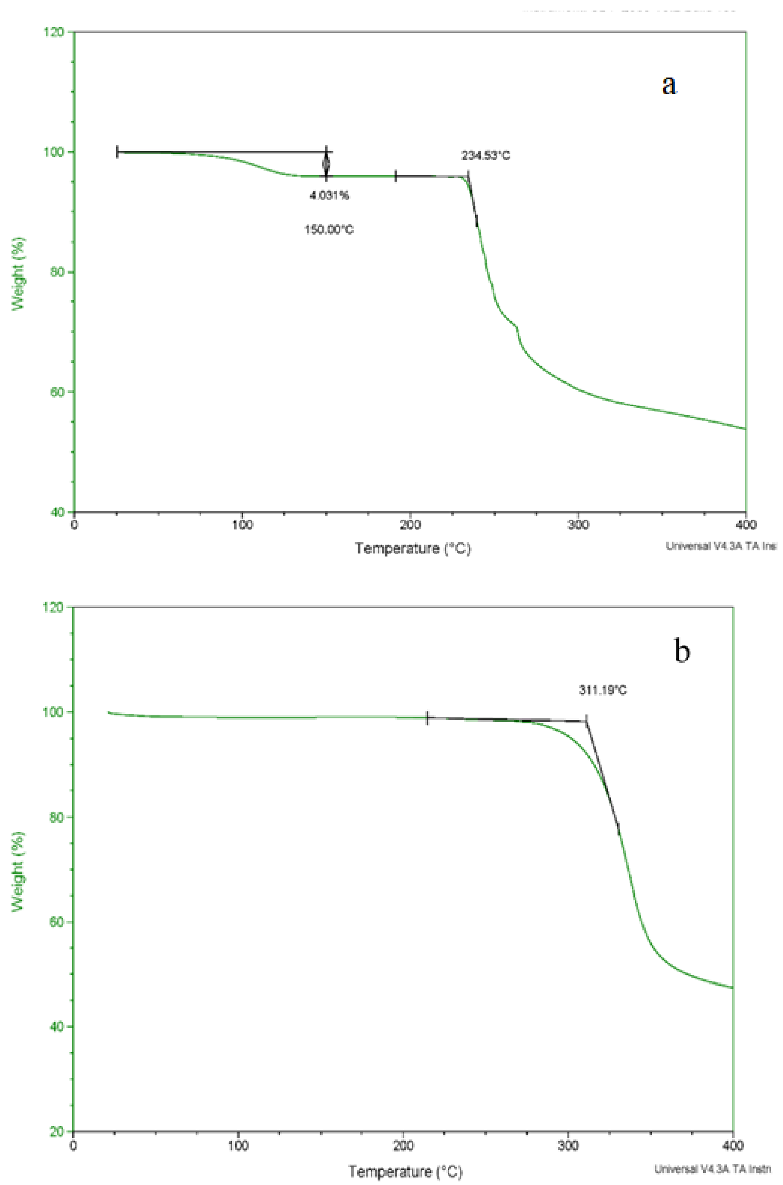

Figure S7. Thermal gravity (TG) curves of (a) EGCG and (b) PEGCG.

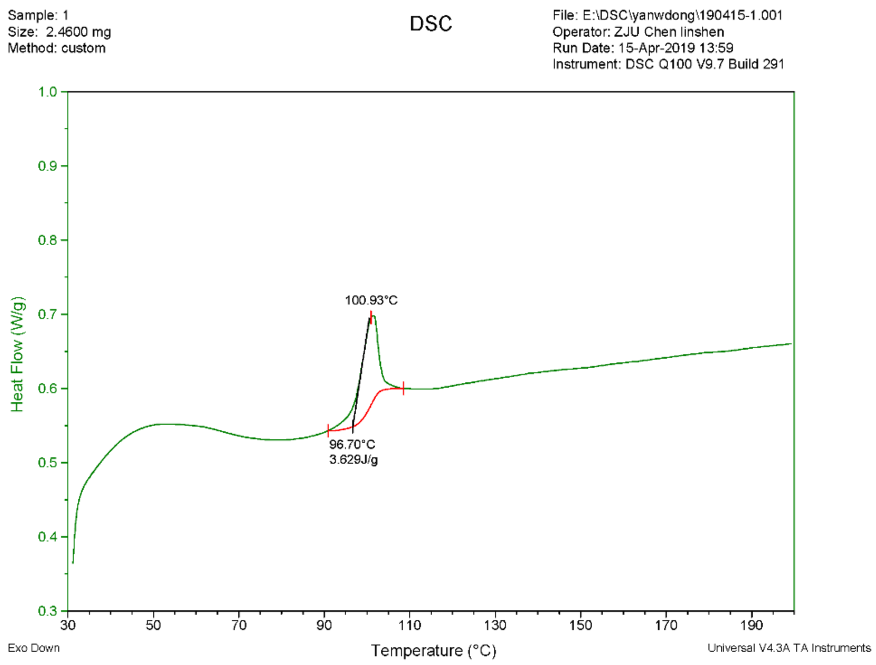

Figure S8. Differential scanning calorimetry (DSC) analysis of PEGCG.

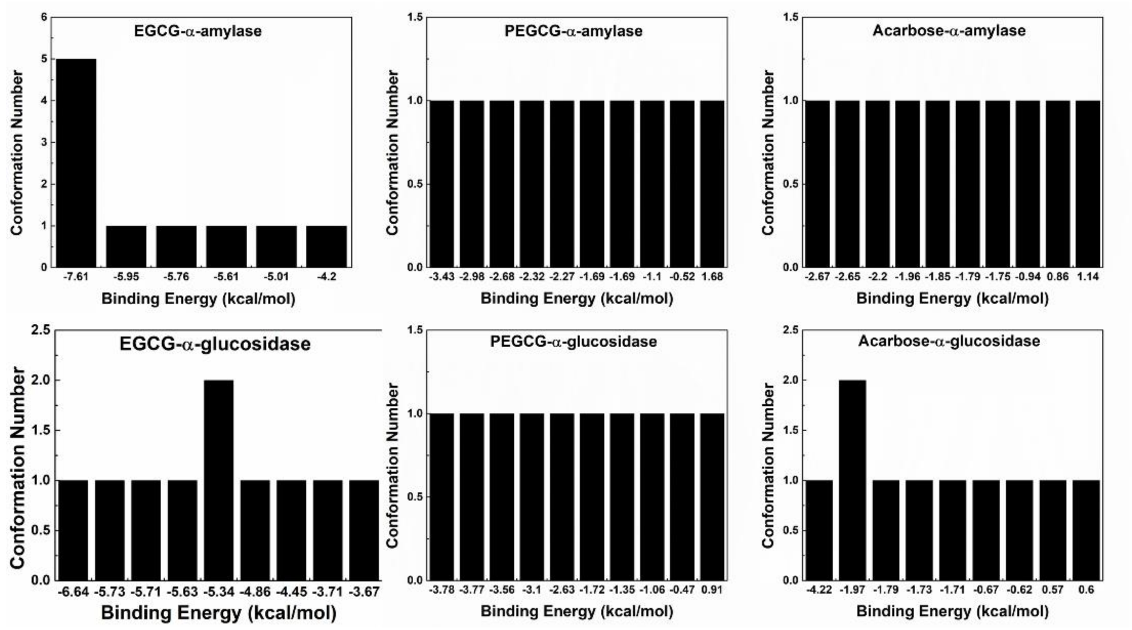

Figure S9. Conformation distribution. Ten docking conformations of each ligand are clustered by binding energy. The RMSD for clustering is set at 0.2 nm.

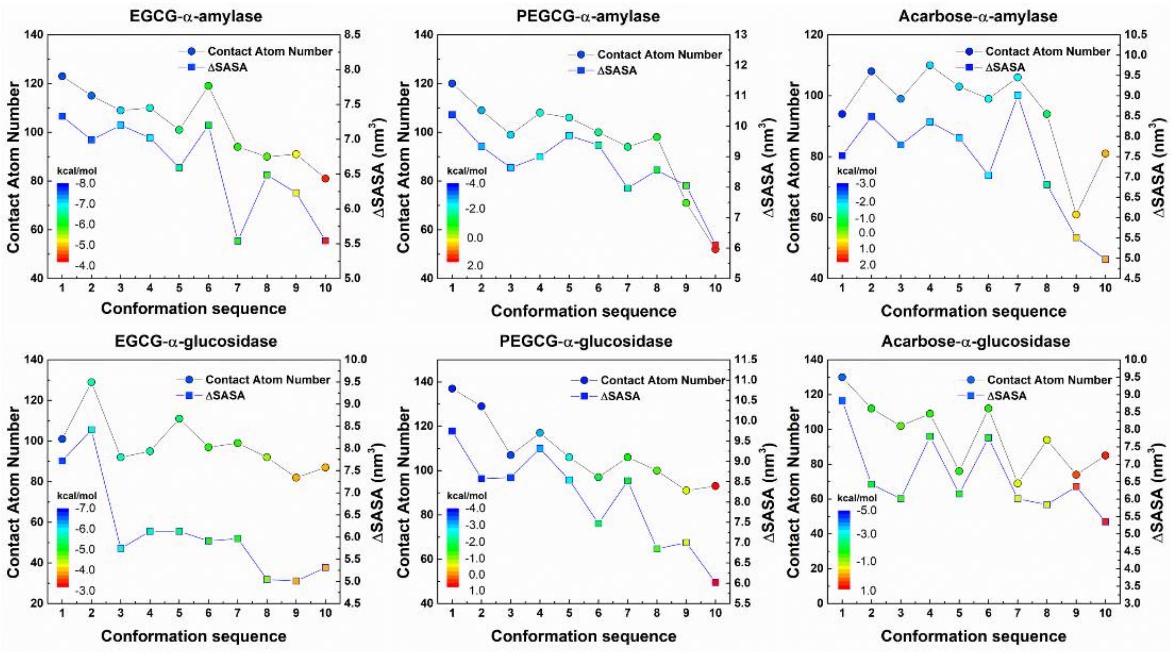

Figure S10. Contact atom number, contact area and binding energy of ten docking conformations. The contact atom numbers are calculated by counting the atom numbers of protein around 0.5nm of ligand. Solution accessible surface area between ligand and protein are calculated for contact area. Cubes and circles colored by binding energy represent contact atom numbers and contact areas, respectively.

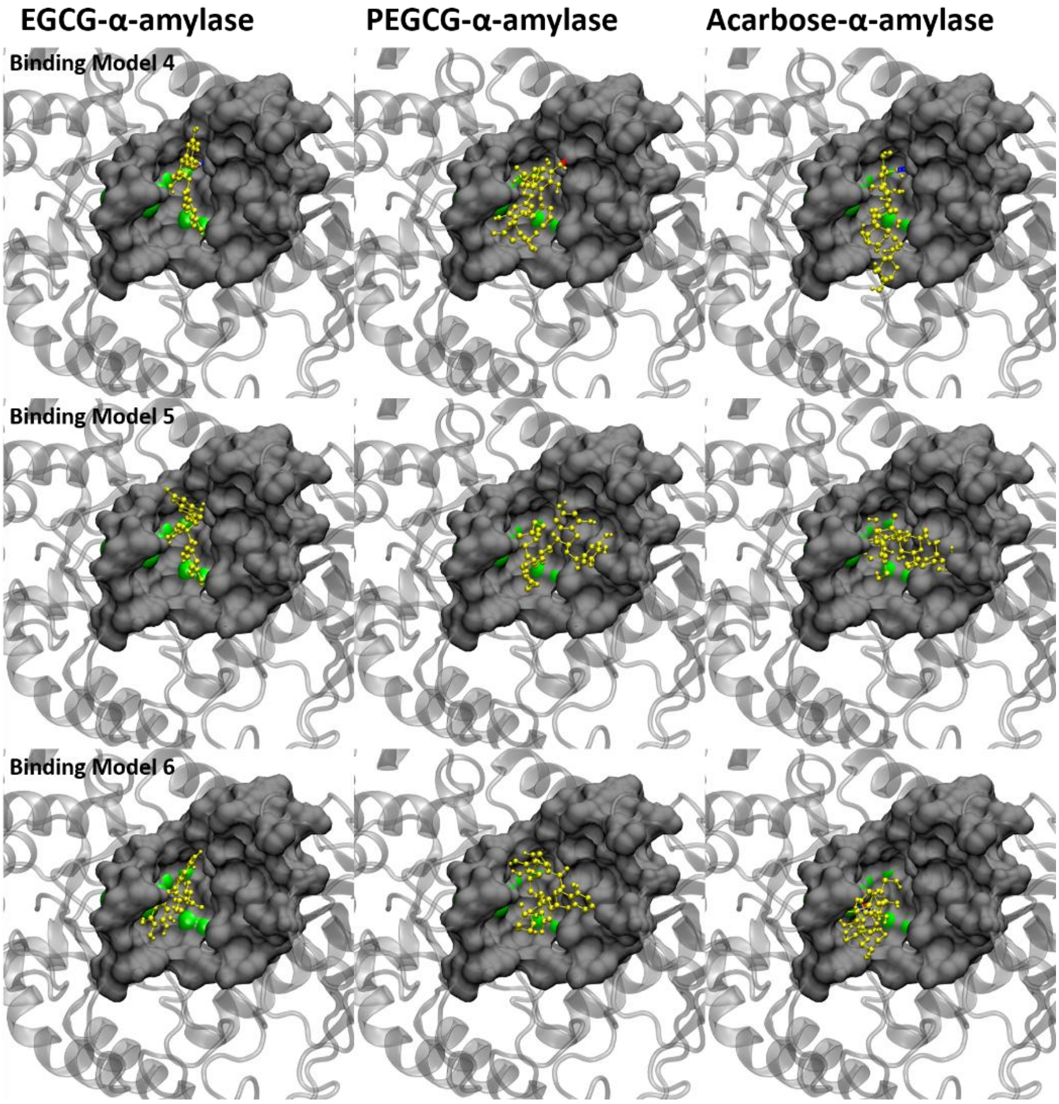

Figure S11. Predicted binding models 4 to 6 of the ligands with α-amylase. Protein is shown in silver cartoon model. Drugs are shown in yellow. The catalytic sites are displayed in green. Amino acids in contact are displayed in surface model. The red and blue dotted lines stand for hydrogen bonds.

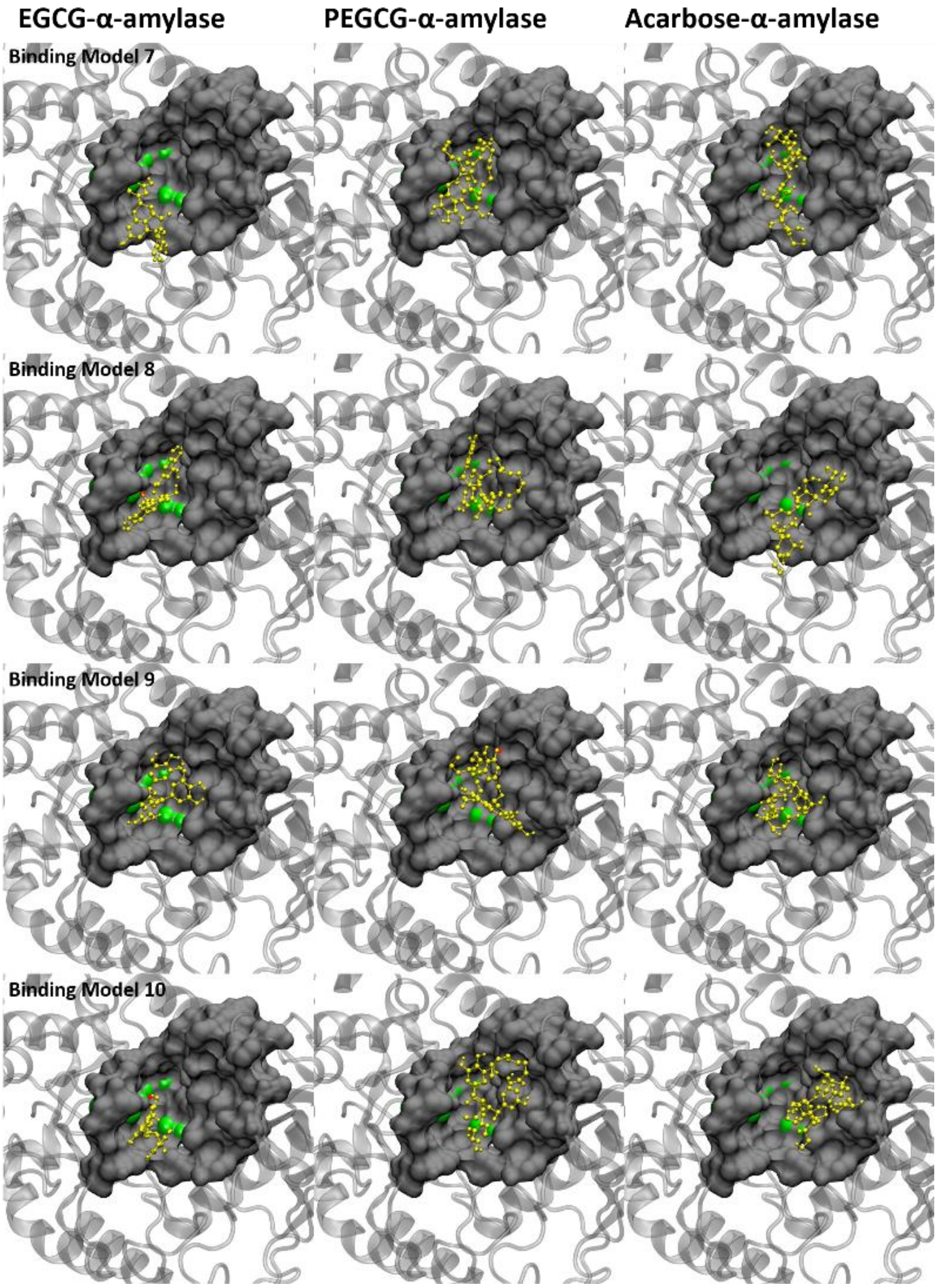

Figure S12. Predicted binding models 7 to 10 of the ligands with α-amylase. Protein is shown in silver cartoon model. Drugs are shown in yellow. The catalytic sites are displayed in green. Amino acids in contact are displayed in surface model. The red and blue dotted lines stand for hydrogen bonds.

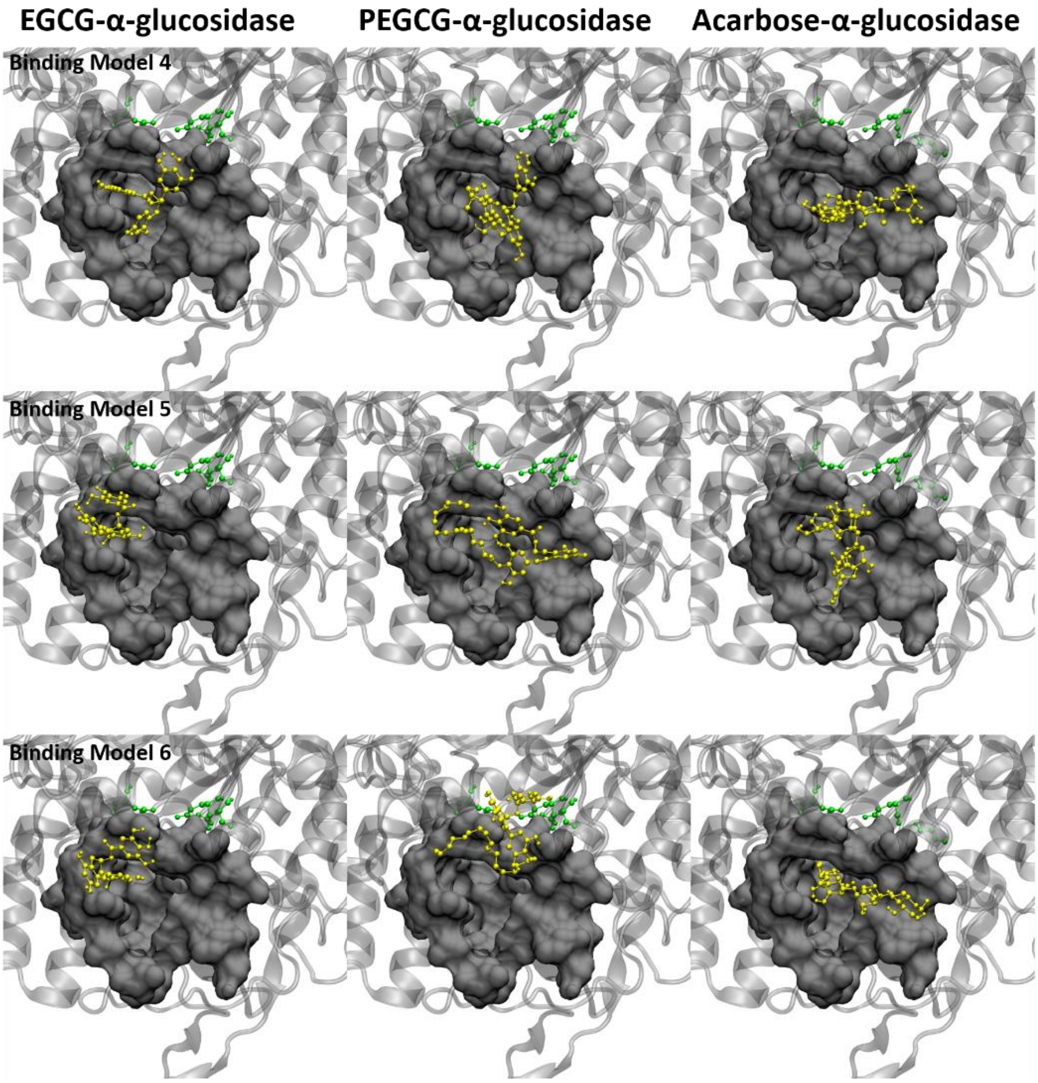

Figure S13. Predicted binding models 4 to 6 of the ligands with α-glucosidase. Protein is shown in silver cartoon model. Drugs are shown in yellow. The catalytic sites are displayed in green. Amino acids in contact are displayed in surface model. The red and blue dotted lines stand for hydrogen bonds.

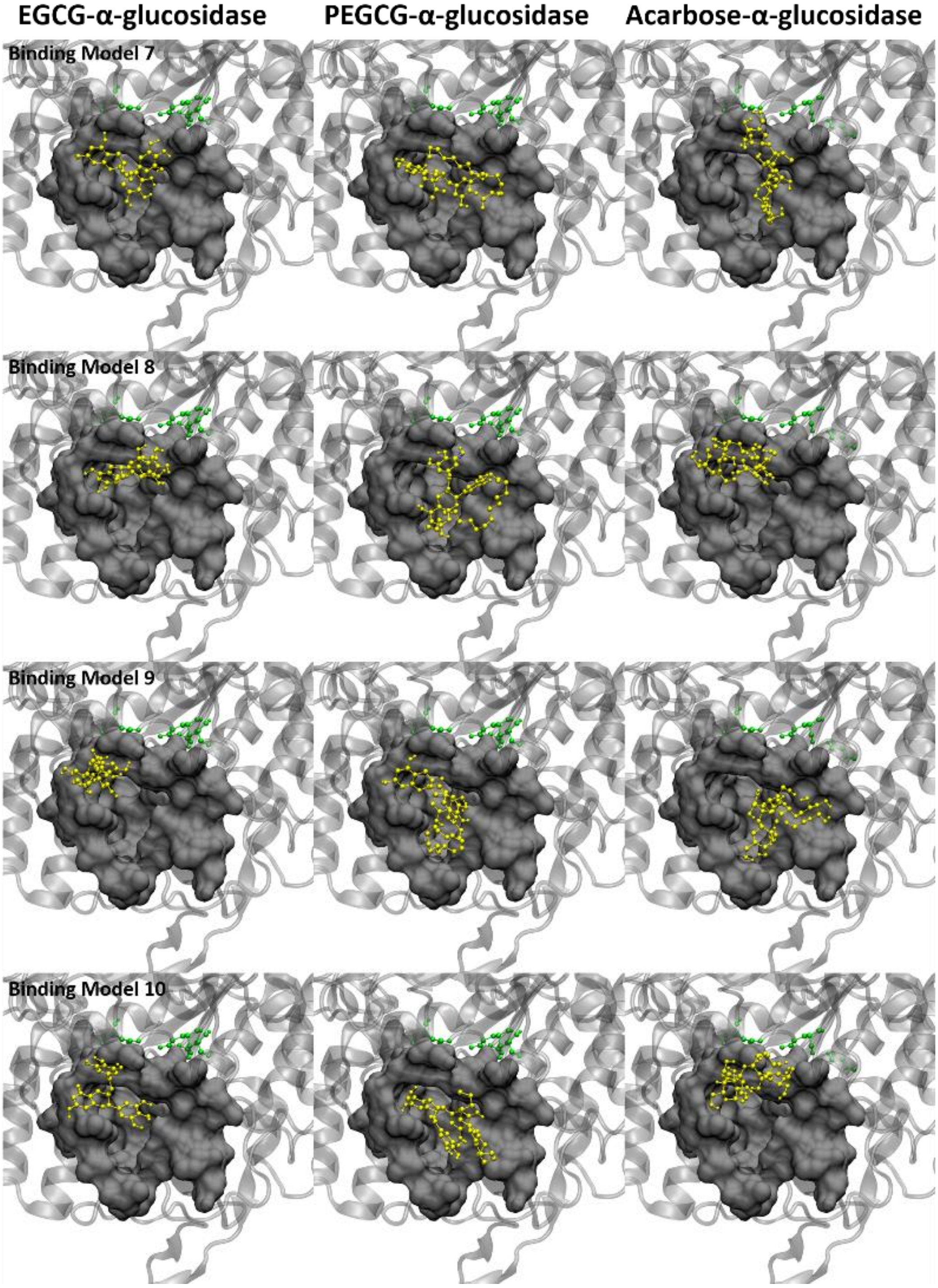

Figure S14. Predicted binding models 7 to 10 of the ligands with α-glucosidase. Protein is shown in silver cartoom model. Drugs are shown in yellow. The catalytic sites are displayed in green. Amino acids in contact are displayed in surface model. The red and blue dotted lines stand for hydrogen bonds.

Table S1. Binding details of the ligands with α-amylase.

α-Amylase	EGCG	PEGCG	Acarbose
Number of contact residues	20	22	19
Residues around 0.5 nm of ligands	TRP13	TYR56	TRP13
	TYR56	PRO122	TYR56
	GLU189	ARG125	TYR193
	PHE190	ASP164	LEU196
	TYR193	ASN172	MET197
	LEU196	ILE174	ARG229
	ARG229	PHE190	ASP231
	ASP231	TYR193	ALA232
	ALA232	TYR195	VAL233
	VAL233	LEU196	LYS234
	LYS234	MET197	HIS235
	HIS235	TYR198	GLU261
	GLU261	ASP231	TYR262
	TYR262	ALA232	TRP263
	TRP263	LYS234	HIS327
	HIS327	HIS235	ASP328
	ASP328	GLU261	GLN333
	GLN333	TRP263	SER334
	SER334	ASP328	LEU335
	LEU335	SER334	
		LEU335	

		HIS105	
Number of the hydrogen bonds	4	2	4
Hydrogen bonds	ligands-ASP328		ligands-ASP328
	ligands-LYS234		ligandsLYS234
	ligands-GLU261	ligands-ARG125	ligands-GLU261
	ligands-GLU261	ligands-TYR193	ligands-GLU261

Table S2. Binding details of the ligands with α-glucosidase.

α-Glucosidase	EGCG	PEGCG	Acarbose
Number of contact residues	19	27	26
Residues around 0.5 nm of ligands	SER157	LYS156	LYS156
	TYR158	SER157	SER157
	PHE159	TYR158	TYR158
	GLY160	GLN239	SER240
	VAL232	SER240	ASP242
	SER240	SER241	LEU246
	ASP242	ASP242	ASN247
	HIS280	PRO243	GLN279
	ASP307	TYR244	HIS280
	THR310	THR245	ALA281
	SER311	LEU246	SER282
	PRO312	ASN247	LYS286
	LEU313	HIS280	ASN302
	PHE314	ALA281	PHE303
	ARG315	SER282	SER304
	TYR316	ASP283	ASP307
	GLU411	THR285	THR310
	ASN415	LYS286	SER311
	ARG442	ASN302	PRO312
		SER304	LEU313
		ASP307	PHE314
		SER311	ARG315
		PRO312	TYR316
		LEU313	GLU332
		PHE314	GLU411
		ARG315	ASN415
		GLU332	
Number of the hydrogen bonds	3	4	5
Hydrogen bonds	ligands-THR310	ligands-GLU332	ligands-THR310
	ligands-ASN415	ligands-HIS280	ligands-ARG315
	ligands-TYR158	ligands-GLU332	ligands-HIS280
		ligands-ASP242	ligands-GLU332
			ligands-ARG315
